# Identification of novel anelloviruses with broad diversity in UK rodents

**DOI:** 10.1099/vir.0.065219-0

**Published:** 2014-07

**Authors:** Shoko Nishiyama, Bernadette M. Dutia, James P. Stewart, Anna L. Meredith, Darren J. Shaw, Peter Simmonds, Colin P. Sharp

**Affiliations:** 1 The Roslin Institute and Royal (Dick) School of Veterinary Studies, University of Edinburgh, Easter Bush Campus, Midlothian EH25 9RG, UK; 2 Department of Infection Biology, University of Liverpool, Liverpool Science Park, 146 Brownlow Hill, Liverpool L3 5RF, UK

## Abstract

Anelloviruses are a family of small circular ssDNA viruses with a vast genetic diversity. Human infections with the prototype anellovirus, torque teno virus (TTV), are ubiquitous and related viruses have been described in a number of other mammalian hosts. Despite over 15 years of investigation, there is still little known about the pathogenesis and possible disease associations of anellovirus infections, arising in part due to the lack of a robust cell culture system for viral replication or tractable small-animal model. We report the identification of diverse anelloviruses in several species of wild rodents. The viruses are highly prevalent in wood mice (*Apodemus sylvaticus*) and field voles (*Microtus agrestis*), detectable at a low frequency in bank voles (*Myodes glareolus*), but absent from house mice (*Mus musculus*). The viruses identified have a genomic organization consistent with other anelloviruses, but form two clear phylogenetic groups that are as distinct from each other as from defined genera.

## Introduction

Human infections with small ssDNA viruses of the family *Anelloviridae* are now considered to be virtually ubiquitous (Hino & Miyata, 2007; Simmonds *et al.*, 1999). The prototype anellovirus, human torque teno virus (TTV), was originally reported in 1997 in a Japanese patient with post-transfusion hepatitis of unknown aetiology (Nishizawa *et al.*, 1997). Since that initial discovery, a large and diverse population of human anelloviruses has been characterized. There are currently five genogroups of TTV, as well as two separate viral genera containing viruses with similarities in genomic organizations but smaller genome size and a virtual absence in identifiable sequence homology throughout large parts of the genome designated torque teno midi virus (TTMDV) (Jones *et al.*, 2005; Ninomiya *et al.*, 2007a, b) and torque teno mini virus (TTMV) (Takahashi *et al.*, 2000). In addition, there has been a large number of anellovirus species reported in wild and domesticated animals, including pigs, wild boar, camels, cats, dogs, pine martens, badgers, sea lions and a number of non-human primates (Abe *et al.*, 2000; Al-Moslih *et al.*, 2007; Martínez *et al.*, 2006; Ng *et al.*, 2009b; Okamoto *et al.*, 2001b, 2002; Romeo *et al.*, 2000; Thom *et al.*, 2003; van den Brand *et al.*, 2012; Verschoor *et al.*, 1999).

Sequence variability within the anelloviruses is extremely high at both the nucleotide and amino acid levels. Divergence, even in the relatively conserved ORF1, is ~50 % at the amino acid level between the five major genogroups of human TTV and variation of >70 % is observed between different genera (Biagini, 2009). Furthermore, humans may at any one time be viraemic with multiple genotypes of TTV, TTMDV and TTMV (Biagini *et al.*, 2006a, b; Moen *et al.*, 2002; Ninomiya *et al.*, 2008). Although the high degree of sequence variation present in TTV and related anelloviruses can pose technical problems for reliable detection by PCR or other amplification methods, viraemia frequencies in human populations of up to 90 % have been reported (Hino & Miyata, 2007; Kakkola *et al.*, 2002).

Despite the almost ubiquitous nature of anelloviruses in human and other mammalian populations, there remain a large number of unanswered questions regarding these infections. Is there any true disease process or identifiable pathology? What are the host cell responses to infection? What are the functions of the encoded viral proteins? How does the virus replicate? The difficulty in obtaining answers to these and other questions stems, in part, from the lack of suitable *in vitro* and *in vivo* model systems for viral replication. Several attempts to replicate both human and porcine TTVs *in vitro* have been unsuccessful (Huang *et al.*, 2012; Kakkola *et al.*, 2007). When *in vitro* replication has been reported (de Villiers *et al.*, 2011), propagation of infection has been complicated by a decline of viral replication following serial passage and an increasing production of subviral molecules.

Experimental infection of laboratory animals including primates (Tawara *et al.*, 2000) with human TTVs has been performed but, to date, pigs have proven to be the best model species in which to study anellovirus infections. Natural infections with viruses from the two genera known to infect pigs [torque teno sus virus 1 (TTSuV1) and torque teno sus virus 2 (TTSuV2)] are common worldwide, with prevalence rates of 24–100 % reported (Bigarre *et al.*, 2005; Gallei *et al.*, 2010; Kekarainen *et al.*, 2006; McKeown *et al.*, 2004; Taira *et al.*, 2009). Experimental infections of gnotobiotic pigs with plasma from conventional TTSuV1-positive animals resulted in viraemia in the recipient animals when tested 28 days post-inoculation (Krakowka & Ellis, 2008; Krakowka *et al.*, 2008), and subsequent inoculation of liver/bone marrow homogenates from these infected animals was also able to transmit the infection to naïve piglets (Mei *et al.*, 2011). More recently, successful rescue of clonal virus by *in vivo* transfection of dimerized genome-containing plasmids directly into piglet lymph nodes has been reported (Huang *et al.*, 2012) and this model is likely to prove invaluable in the future. Development of a rodent model would provide a number of advantages over large-animal models because of the wide array of reagents and resources available for mice, as well as the financial and technical benefits of study in a small species. However, attempts to develop a reliable rodent model by infecting laboratory mice with human TTV have not proven successful (Isaeva & Viazov, 2002) and the use of viruses naturally adapted to murine hosts may be required.

Given the high rates of anellovirus infections in humans and their presence in other mammalian species, it would be predicted that these infections would also be present in mice or other members of the order Rodentia. However, no such infection has been reported previously from either laboratory or wild rodent populations. Factors contributing to the lack of reported detection in laboratory mice may include the high degree of sequence variability, even in the conserved UTR, precluding PCR-based approaches described for human, primate and other animal screening. Furthermore, the sterile conditions under which laboratory mice are kept may effectively prevent transmission of infection by natural routes. In this study, we therefore concentrated screening efforts towards wild rodent populations, including wood mice (*Apodemus sylvaticus*), field voles (*Microtus agrestis*), bank voles (*Myodes glareolus*) and house mice (*Mus musculus*) from the UK using a sequence-independent rolling circle amplification (RCA) method combined with traditional restriction digest/cloning that has previously been applied for the identification of novel circular DNA viral species (Ng *et al.*, 2009a; Rosario *et al.*, 2012) including anelloviruses (Breitbart & Rohwer, 2005; Cornelissen-Keijsers *et al.*, 2012; Ng *et al.*, 2009b). Characterization of cloned complete genomes of TTV-like viruses detected in these rodent species allowed us to assess the variability of these viruses, both within and between host species, and to design a novel PCR-based screening method with which to assess the prevalence and molecular epidemiology of TTV-like viruses in larger rodent populations.

## Results

### Identification of novel viral sequences in wild rodent DNA samples

In order to screen wild rodent populations for anelloviruses, six RCA libraries were constructed: two from wild house mice spleens, two from wild wood mice sera, and one each from a wild wood mouse spleen and a laboratory mouse spleen. A number of colonies from each library were screened by PCR using plasmid-specific primers for the amplification of inserted sequences (Table 1). Amplicons were sequenced and classified based on blastn homology search results. A total of four candidate anellovirus clones were detected. The two candidate viral clones in the wood mouse WM1 spleen sample contained an identical sequence of 71 nt with a high degree of similarity (*E* = 4×10^−15^) to an uncultured anellovirus database entry (GenBank accession number HQ335012.1). Two sequences from the serum-derived library from the same mouse also had identical sequences of 86 nt with low similarity (*E* = 1.3) to a TTMV isolate (GenBank accession number AB303565.1) and a TTV sequence from a macaque (GenBank accession number AB041958.2). These four sequences were provisionally designated rodent TTV (RoTTV).

**Table 1.** Sequence library screening results

	Wood mouse	Wood mouse	Wood mouse	House mouse	House mouse	House mouse
Sample	Spleen	Serum	Serum	Spleen	Spleen	Spleen
ID Number	WM1	WM1	WM4	WM2	WM3	LM1
Library colonies screened	4	46	12	37	14	17
Anellovirus-like colony sequences [*n* (%)]	2 (50)	2 (4)	0 (0)	0 (0)	0 (0)	0 (0 )

Using opposite polarity primers based on these sequences (RoTTV599inv and RoTTV664inv primers; Table S1, available in the online Supplementary Material), the entire remaining genome was amplified by PCR using the RCA template from the original source samples. The amplicons were then cloned and sequenced. Construction and analysis of these two genome clones (RoTTV599 from spleen and RoTTV664 from serum, later referred to as AS_WM1_Sp_1 and AS_WM1_Se_1, respectively) showed that they were of a size consistent with previously described TTMV genomes (2237 and 2235 nt) and resembled known anelloviruses in genomic organization.

### Wild rodent population screening

The RoTTV599 and RoTTV664 genome clones were found to have sequences in the UTR that were partially conserved in previously described anelloviruses. Primers based on these sequences (Thom *et al.*, 2003) were modified (PanTTV screening primers, Table S1) to allow for additional polymorphic sites in RoTTV sequences, and used to screen spleen and liver DNA from a total of 271 wild rodent samples by PCR. Samples collected in Edinburgh (10 wood mice and two house mice) and rural sites in Scotland (Pentlands and the Borders; 48 wood mice) and England (Cumbria and Cheshire; 67 wood mice, 26 house mice, 79 field voles and 39 bank voles) were tested. The identity of all rodent samples was confirmed from mitochondrial DNA sequence to ensure correct species assignment. RoTTV DNA was detected in liver or spleen from 87 % of wood mice [109/125, 95 % confidence interval (CI): 80–93 %], 60 % of field voles (47/79, 95 % CI: 48–70 %), 8 % of bank voles (3/39, 95 % CI: 2–21 %) and none of the wild house mice (0/28, 95 % CI: 0–12 %). Variation in RoTTV prevalence at different sampling sites was tested for in wood mice, where ≥10 individuals from the four different areas were available (Borders *n* = 30, Pentlands *n* = 18, Cheshire *n* = 64 and Edinburgh *n* = 10), but no significant difference was seen.

**Fig. 1.**
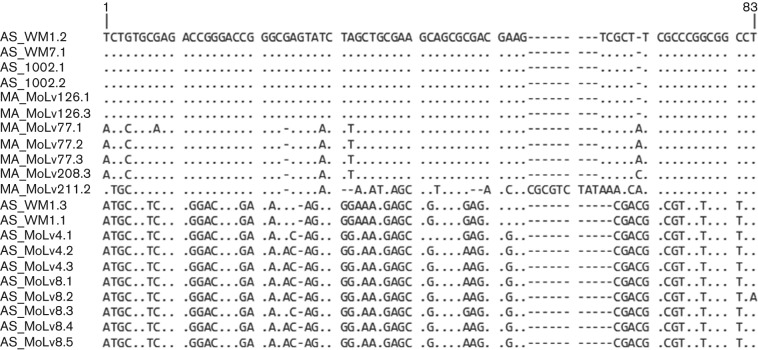
Alignment of cloned RoTTV screening amplicons. Sequences named by host species initials, sample name and clone number.

The PCR amplicons from PanTTV screening were cloned and sequenced for nine animals (five wood mice, three field voles and one bank vole). Following removal of primer sequences, the inserts were found to measure 71–78 bp and showed substantial sequence divergence (Fig. 1). Based on this region, sequences were tentatively classified into two major sets for the purpose of designing the inverted primers for complete genome amplification: RoTTV1 and RoTTV2. RoTTV1 was further classified into three subsets: a (represented by AS_WM1.2), b (represented by MA_MoLv77.1) and c (MA_MoLv211.2).

Given the generally high detection frequency of anellovirus sequences in wood mice and the two vole species, the lack of detection in house mice was unexpected. To broaden the testing of this species, the source of house mice tested was expanded to include a further 29 samples (four spleen samples from BALB/c strain laboratory mice and 25 liver samples from mouse carcasses sold commercially as snake food). All 29 samples tested negative using the PanTTV PCR assay. To further test the possibility that an anellovirus with a UTR sequence that is not detectable using this assay may have been present, an additional five RCA clone libraries were generated from wild house mice samples. Sequencing of 102 colonies from these libraries revealed no anellovirus matches.

### RoTTV complete genome PCR

Inverse primers, based on common cloned UTR sequences, were designed for the amplification of full-length RoTTV genomes from the four identified sets (RoTTV1a_inv, RoTTV1b_inv, RoTTV1c_inv and RoTTV2_inv primer sets; Table S1). DNA extracted from the liver, spleen, serum and/or faecal supernatants of seven PanTTV PCR-positive animals (three wood mice, three field voles and one bank vole) was amplified by RCA followed by PCR amplification with type-specific primers. Amplicons from the RoTTV1_inv (a, b and c) and RoTTV2_inv PCRs were ~2.2 and 2.5 kpb, respectively. A total of 15 RoTTV1 and 13 RoTTV2 genome clones were fully sequenced and included in the subsequent analysis along with the two original clones (GenBank accession numbers KJ194604–KJ194633). The RoTTV sequences were aligned with published TTV sequences in the UTR and numbered accordingly. In two wood mice, AS_1012 and AS_1014, it was possible to sequence genome clones from both spleen tissue and faecal supernatants. RoTTV1 and RoTTV2 clones generated from the different samples in these animals showed up to 99.8 % identity, indicating that anellovirus sequences found in faeces can be representative of those present in tissue and additionally that RoTTV could potentially be spread by the faecal–oral route.

**Fig. 2.**
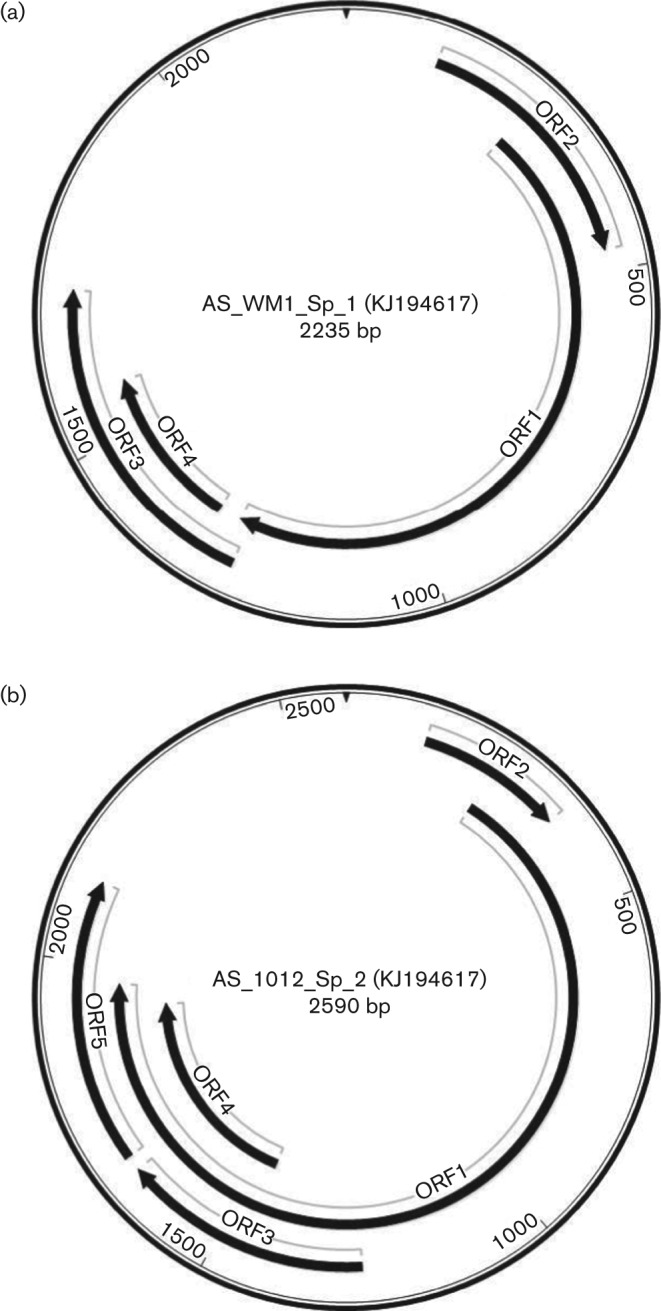
Representative genome maps of (a) RoTTV1 and (b) RoTTV2. GenBank accession numbers are given in parentheses.

A representative genomic map, showing major ORFs, of the 15 RoTTV1 clones and two original clones is shown in Fig. 2(a). Whilst the overall genome layout was conserved among these clones, the overall genome sizes and precise length of the ORFs were variable but could be broadly divided into four groups based on ORF size (Table 2). The translated amino acid sequences encoded by the ORFs positionally equivalent to the ORF2 and ORF1 in other anelloviruses indeed showed identifiable homology to these coding regions, particularly those of TTSuV and mosquito VEM anellovirus. The 123–145 and 83–127 aa sequences encoded by the other major ORFs (designated ORF3 and ORF4, respectively) did not show any identifiable homology to sequences in the protein database, including ORFs from other anelloviruses.

**Table 2.** Genome length and ORF sizes of the 17 RoTTV1 and 13 RoTTV2 complete genome sequences grouped by ORF size

	Genome length (nt)	ORF2 (aa)	ORF1 (aa)	ORF3 (aa)	ORF4 (aa)	ORF5 (aa)
RoTTV1						
Group 1 (*n* = 10)	2236–2317	115	342	145	83	
Group 2 (*n* = 2)	2265	94	346	123	127	
Group 3 (*n* = 4)	2163–2247	110	344	137	86	
Group 4 (*n* = 1)	2263	94	346	143	83	
**RoTTV2**						
Group 1 (*n* = 11)	2590–2591	75	576	128	156	147
Group 2 (*n* = 2)	2591	75	576	83	156	147

A representative genomic map, showing major ORFs, of the 13 RoTTV2 clones is shown in Fig. 2(b). Again, the overall genome layout was conserved among these clones but the overall genome sizes and precise length of the ORFs were somewhat variable and could be broadly divided into two groups based on ORF size (Table 2). As with the RoTTV1 clones, blastp analysis of the translated amino acid sequences encoded by the ORFs positionally equivalent to ORF2 and ORF1 showed close similarity to these coding regions. The other major potential coding regions (designated ORF3, ORF4 and ORF5) showed no identifiable homology to sequences in the protein database and, unlike RoTTV1 ORF3 and ORF4, did not contain a start codon. It is predicted that these ORFs would be expressed through splicing with ORF2 and ORF1 as has been shown in other anelloviruses (Huang *et al.*, 2011; Martínez-Guinó *et al.*, 2011; Mueller *et al.*, 2008).

### RoTTV phylogenetic analysis

**Fig. 3.**
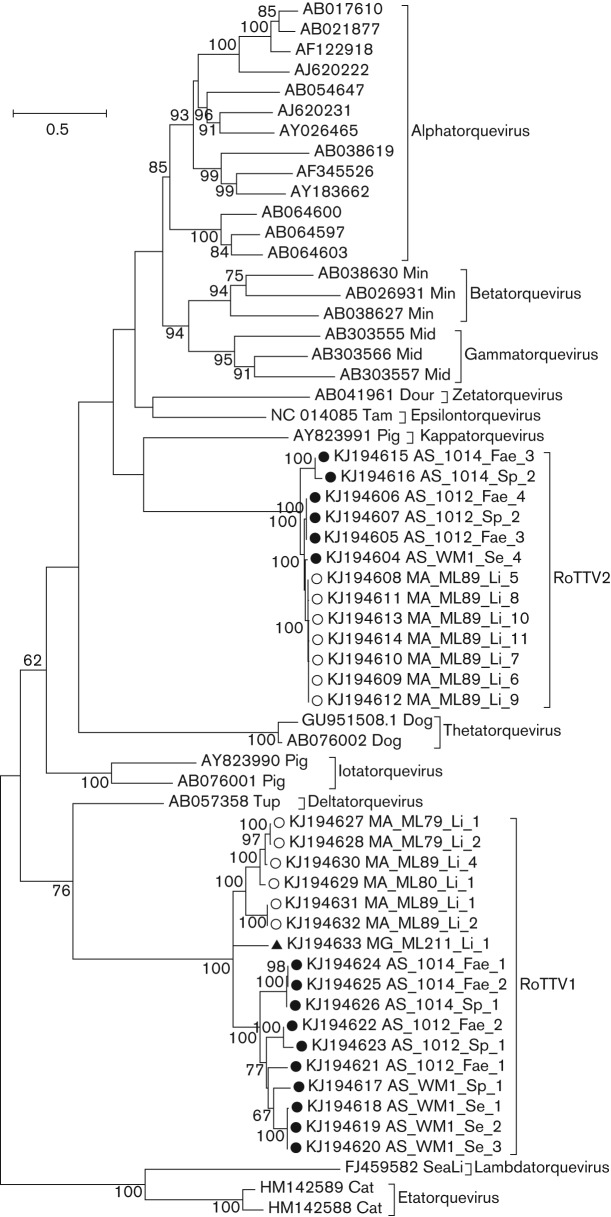
RoTTV phylogeny as inferred from partial ORF1 nucleotide sequences (equivalent to nt 124–723 of the prototype RoTTV sequences AS_WM1_Sp_1). The tree was reconstructed using representative sequences from human TTV, TTMV and TTMDV groups, as well as sequences from the eight non-human anellovirus genera infecting dogs, cats, pigs, douroucoulis (Dour), tamarins (Tam), sea lions (SeaLi) and tupias (Tup). Novel sequences of RoTTVs from wood mice (•), field voles (○) and a bank vole (▴) are marked. The evolutionary history was inferred using maximum-likelihood methods as implemented in the mega6 software package (Tamura *et al.*, 2013). The optimum maximum-likelihood models (lowest Bayesian information criterion score and typically greatest maximum-likelihood value) for the nucleotide sequence alignment was first determined and used for phylogenetic reconstruction. This was the general time reversible model with a γ distribution (five rates) and invariant sites (GTR+γ+I). Bootstrap support of branches (500 replications) is indicated.

The translated RoTTV ORF1 sequences, along with 164 anellovirus ORF1 sequences available in GenBank, were aligned using muscle version 3.8. The aligned nucleotide sequences from the region of highest similarity (equivalent to nt 124–723 of AS_WM1_Sp_1 ORF1) were used for subsequent phylogenetic and pairwise-comparison analysis. A phylogenetic tree containing all RoTTVs and representative sequences from the 11 currently classified anellovirus genera (including human TTV, TTMV, TTMDV and other animal anelloviruses) is shown in Fig. 3. RoTTV1 and RoTTV2 sequences formed two distinct clades, with less variation seen in RoTTV2. Pairwise distance comparisons of the amino acid sequences across the analysed region of all available human and chimpanzee anelloviruses (TTV *n* = 129, TTMV *n* = 14, TTMDV *n* = 21) and RoTTVs (RoTTV1 *n* = 17, RoTTV2 *n* = 13) are shown in Table 3. Sequence divergences of 18.7 and 2.5 % were observed within RoTTV1 and RoTTV2 sequences, respectively. However, a 74.5 % sequence divergence was found between these groups, which was comparable to the difference between these groups and other established anellovirus genera, and was greater than the divergence seen between established genera. Therefore, RoTTV1 and RoTTV2 should be classified at least as distinct species, and the divergence is even enough to potentially classify them as distinct genera pending review by the International Committee on Taxonomy of Viruses.

**Table 3.** Group amino acid pairwise distance comparisons (%) within and among 17 RoTTV1, 13 RoTTV2, 129 TTV, 14 TTMV and 21 TTMDV using partial ORF1 sequences

	RoTTV1 (*n* = 17)	RoTTV2 (*n* = 13)	TTV (*n* = 129)	TTMV (*n* = 14)	TTMDV (*n* = 21)
RoTTV1 (*n* = 17)	18.7	74.5	74.1	77.3	77.7
RoTTV2 (*n* = 13)		2.5	74.3	75	74.6
TTV (*n* = 129)			49.4	68.2	64.6
TTMV (*n* = 14)				54.4	67.5
TTMDV (*n* = 21)					50.1

### Distribution of RoTTV1 and RoTTV2 in wild rodent populations

Based on the complete genome sequences, two sets of specific nested primers were constructed based on sequences distinct between RoTTV1 and RoTTV2 in the ORF1 coding region (RoTTV1_ORF1 and RoTTV2_ORF1 primer sets; Table S1). All wild rodent DNA samples were screened using these specific PCRs (Table 4). RoTTV1 was the more frequently detected virus in wood mice (81 %), but RoTTV2 was also detected frequently (54 %). Co-infection with members of both viral species was common in this host (43 %). RoTTV1 is also the more frequently detected virus in field voles (63 %) with a much rarer detection of RoTTV2 (8 %). In bank voles, where overall prevalence is markedly lower, there was frequent detection of RoTTV2 (15 versus 8 %).

**Table 4.** Number and prevalence (and exact binomial 95 % CIs) of PanTTV and RoTTV1/2 in four species of wild rodents tested by PCR

		Wood mice (*n* = 125)	Field voles (*n* = 79)	Bank voles (*n* = 39)	House mice (*n* = 28)
PanTTV positive	*N*	109	47	3	0
	%	87 (80–93)	59 (48–70)	8 (2–21)	0 (0–12)
RoTTV1 positive	*N*	101	50	3	0
	%	81 (73–87)	63 (52–74)	8 (2–21)	0 (0–12)
RoTTV2 positive	*N*	67	6	6	0
	%	54 (44–63)	8 (3–16)	15 (6–31)	0 (0–12)
RoTTV1/2 co-infection	*N*	54	5	1	0
	%	43 (34–52)	6 (2–14)	3 (0–13)	0 (0–12)

A total of 21 samples (10 wood mice, six bank voles and five field voles) were positive using the type-specific PCRs that were previously negative with the PanTTV PCR. In these cases, it was likely that the higher detection rate of the type-specific primers allowed for the detection of lower-titre viruses. In seven samples that were previously found to be PanTTV-positive (five wood mice, one bank vole and one field vole), no detection of RoTTV1 or RoTTV2 could be found, suggesting that further divergent RoTTVs may be present.

## Discussion

The amplification of viral genomes by RCA has become increasingly widely used since its first reported application in 2004 (Inoue-Nagata *et al.*, 2004) and has been found to be particularly useful in cases where cell culture cultivation of viruses is not possible. In recent years, this amplification technique has been combined with traditional restriction digest/cloning (Ng *et al.*, 2009a; Rosario *et al.*, 2012) or next-generation sequencing (Rosario *et al.*, 2009) to provide information on viral metagenomes, leading to the identification of several novel viral species, and has been widely used in the identification and characterization of anellovirus populations (Biagini *et al.*, 2007; Cornelissen-Keijsers *et al.*, 2012; Macera *et al.*, 2011; Ng *et al.*, 2009b; van den Brand *et al.*, 2012). In this study, we used RCA combined with traditional restriction digest/cloning to identify novel anellovirus sequences in the spleen tissue and serum of wild rodents. Rather than use filtration combined with DNase treatment to enrich for encapsidated viral genomes, we employed a size-fractionation protocol where total DNA was extracted from tissue and separated by gel electrophoresis. From our results, it can be seen that this technique is suitable for the enrichment of small circular viral genomes; however, due to degradation of host DNA in some cases, we were limited to viruses that ran further than linear fragments of ~3000 bp where there could be clear separation from the host DNA. Although this fractionation may have excluded some larger viruses, it allowed for the screening of fewer clones and it should be noted that no larger viral genomes were detected in the serum DNA libraries where no size fractionation step was used. The *Bst*UI restriction enzyme was chosen for the digestion of RCA-amplified DNA as it has a four-base recognition site and should therefore cut more frequently than more commonly used six-base cutters (approximately once every 256 bp versus once every 4096 bp in a random sequence). Whilst lacking the depth of coverage provided by next-generation sequencing, we have clearly supported previous findings that, with appropriate sample enrichment, this method has the ability to identify such novel sequences following screening of a modest number of colonies and at a fraction of the cost.

Following complete genome sequencing, rodent anellovirus genomes were found to be smaller than those of human TTV. Genome sizes of 2.2–2.5 kb were more comparable to human TTMV and to genomes of other non-primate anelloviruses that typically range from 2 to 3 kb in size. Despite the size variation evident in anellovirus genomes, RoTTV genome sequences reported here showed clear homology to known anelloviruses, not only in ORF1 and ORF2 sequence, but in overall genomic organization. The conservation of distinct motifs within the UTR allowed for the use of modified universal anellovirus primers to determine the prevalence of TTV-like viruses, both closely and more distantly related to the initially identified clones, in the wider wild rodent population. A detection frequency of 87 % in wild wood mice was comparable to those in humans (>90 %) and other wild mammalian populations tested, including wild boar (84 %) (Martínez *et al.*, 2006) and pine martens (100 %) (van den Brand *et al.*, 2012). The 60 % detection frequency in field voles was similarly high, but a level of detection of only 8 % was seen in bank voles. It should be noted that, in contrast to the wood mice, samples from these species were tested from sites in a single area and the frequencies found may not be representative of the wider population. We also acknowledge the difficulty in designing effective screening methods for such divergent viruses and that this latter detection rate may have originated from infection of the latter species with TTV variants that were sufficiently divergent to preclude effective amplification. However, it is also possible that natural infection in wild populations does not inevitably lead to very high prevalence rates. Differences in ecology preventing spread at the population level may exist in these species or the individuals possess some form of resistance – either a barrier to initial infection, poor transmission or an ability to clear virus. Further work, including the development of a serological assay to look for anti-RoTTV antibodies in these species, would be needed to address these questions.

The complete absence of anelloviruses in 28 wild house mice was also unexpected and indicates that anellovirus infection, if present, occurs at a lower frequency in house mice than in the other rodent species tested. However, based on the relatively small sample number, it is possible that a prevalence of infection of up to 12 % could still be present with 95 % confidence. To differentiate low prevalence from absence of infection, screening of a much larger sample set would be required. Discrepancies between infection rates in UK wild wood and house mice, similar to those seen here for RoTTV, have been reported with another virus, murid herpesvirus 4 (Blasdell *et al.*, 2003), and the parasite *Toxoplasma gondii* (Thomasson *et al.*, 2011). As for bank voles, it is possible that anelloviruses are present in house mice but are either too divergent for detection by PCR or alternatively have features such that our cloning methodologies were insufficient for their identification, e.g. a low population frequency, low titre or absence of recognition sites for the *Bst*UI restriction enzyme in the genome. If there is indeed an absence of a house mouse anellovirus, this may be related to the fact that house mice are not native to the UK and have only been introduced in the last few hundred years (Auffray *et al.*, 1988; Lundrigan *et al.*, 2002; Morse, 1981). This introduction may have caused a population bottleneck where house-mice-specific anelloviruses were lost. Alternatively, there may be a fundamental difference in the biology, particularly immunology, of house mice that renders them truly resistant to infection.

This study has shown that RoTTV infections have many features in common with human anellovirus infections: (i) there is a high rate of infection, at least in the wood mouse and field vole species; (ii) the presence of multiple genotypes of virus or even multiple species/genera can be found in a single individual at a given time; and (iii) the presence of viral DNA can be found in multiple sites in an individual. Our population studies focussed on detection in spleen and liver tissue. Although these tissues are generally not currently considered as sites of viral replication, their use for the detection of anellovirus DNA has been well documented (Aramouni *et al.*, 2010; Okamoto *et al.*, 2001a) and may be due primarily to the presence of circulating virus in these highly vascularized organs. The detection of virtually identical viruses in tissue and the faecal samples of individuals supports the potential for faecal–oral virus transmission (Lin *et al.*, 2000). Faecal samples and rectal swabs have also been used in the identification of novel anelloviruses, and this finding supports the conclusion that these viruses do represent infections of the animals studied (pine martens and badgers), although a dietary source cannot be completely ruled out (van den Brand *et al.*, 2012).

Studies into the basic virology, pathogenesis and disease associations of human TTV have been limited by a number of complicating factors, including the high degree of genetic variability leading to many false-negative tests, the difficulty in identification of negative control populations and the lack of a robust cell culture system for viral replication. Given the broad range of laboratory reagents and background knowledge available for studies in rodents, we believe that RoTTV will prove a valuable resource for anellovirus research. Should experimental infection of the well-studied laboratory mouse strains (*Mus musculus*) prove to be unproductive, it should still be possible to experimentally infect laboratory-bred wood mice to give a tractable animal model. Such infections would allow for the direct investigation into a number of unanswered or contentious questions in anellovirus infection, including transmission routes, persistence, viral species differences, pathogenesis and disease associations.

## Methods

### 

#### Samples.

Wild rodent samples were collected in three rural study sites in northern England (Cumbria) and Scotland (Pentlands and Borders) as part of DEFRA (Department for Environment, Food and Rural Affairs) project SE01526 (Meredith *et al.*, 2013), and in Cheshire (England) as part of previous studies on rodent herpesviruses (Ehlers *et al.*, 2007; Hughes *et al.*, 2010). Further wild rodent samples were captured at sites within Edinburgh. Wild rodents were captured in humane traps and euthanized by overdose of volatile anaesthetic (isoflourane) and cervical dislocation. Outbred *Mus musculus* were purchased frozen as commercially available pet food.

#### Nucleic acid extraction.

Brain, spleen and serum samples from rodents were digested using Proteinase K (final concentration 2 mg ml^−1^) in lysis buffer (4 % SDS, 0.5 M Tris, 0.25 M EDTA, 2.5 M NaCl) at 53 °C overnight. Total nucleic acids from crude lysates were extracted using standard phenol : chloroform : isoamyl alcohol extraction methods and concentrated using ethanol precipitation. Samples were carefully resuspended in TE buffer using broad-ended tips and avoiding vortexing to minimize shearing of genomic DNA. For the extraction of DNA from faecal samples, three to five faecal pellets were first incubated with 200 µl PBS, and mixed by inversion and vortexing for 5 min. Samples were pelleted by centrifugation at 15 000 ***g*** for 5 min. DNA was extracted from the supernatants using the QIAamp DNA Blood Mini kit (Qiagen) following the manufacturer’s instructions.

#### Size fractionation of tissue nucleic acids.

Size fractionation was performed on freshly extracted nucleic acid samples or on samples that had undergone a single freeze–thaw cycle to minimize shearing of genomic DNA. Electrophoresis of 1–5 µg samples of total nucleic acid extracted from brain and spleen tissues in 0.7 % agarose gels was used for size fractionation and confirmation of genomic DNA integrity. Gel slices corresponding to DNAs of ~500–3000 bp were excised from gels following fractionation. DNA was extracted from the gel slices using the QIAquick Gel Extraction kit (Qiagen) according to the manufacturer’s instructions. To prevent contamination, all samples were fractionated on individual gels with fresh buffer in an electrophoresis tank that was cleaned thoroughly with 10 % MicroSol3 solution and rinsed with distilled H_2_O between runs.

#### RCA.

RCA was performed using the Repli-G Mini kit (Qiagen) according to the manufacturer’s instructions, with the exceptions that Phi-29 polymerase (NEB) was used in place of the supplied enzyme, as we had previously observed marginally better amplified product yield with the NEB enzyme, and the denaturation step prior to amplification was excluded to select for ssDNAs. Between 10 and 100 ng total nucleic acid extracted from serum or size-fractionated nucleic acid from spleen or brain tissue was used as template for the RCA reactions.

#### Cloning of amplified samples.

RCA DNA samples were digested with *Bst*UI restriction endonuclease (NEB) for 4 h and terminal phosphates were removed using Antarctic phosphatase (NEB) according to the manufacturer’s instructions. Digested fragments were ligated into the pCR-Blunt II-TOPO vector (Life Technologies) and these ligation reactions were used to transform TOP 10 F′ chemically competent *Escherichia coli* which were plated on LB/Agar plates supplemented with 50 µg kanamycin ml^−1^. Amplicons from PCRs were ligated into the pGEM-T Easy vector (Promega) and these ligation reactions were used to transform TOP 10 F′ chemically competent *E. coli*, which were plated on LB/Agar plates supplemented with 100 µg ampicillin, 0.5 µM IPTG and 80 µg X-Gal ml^−1^.

#### PCR.

Nested and hemi-nested PCRs for RoTTV ORF1 and PanTTV screening as well as single-round PCR for species identification were performed using GoTaq DNA polymerase (Promega) and primers listed in Table S1. Reactions were performed using 0.5 µg samples of extracted nucleic acid as template under the following conditions: 30 cycles of 18 s at 94 °C, 21 s at 50 °C and 60 s at 72 °C, and a final extension of 5 min at 72 °C.

Full-length PCR were carried out using AccuPrime *Taq* High Fidelity (Life Technologies). Reactions were performed using 10× AccuPrime PCR Buffer I, 1 µl amplified DNA by RCA as template and the inverted primers shown in Table S1, and cycled under the following condition: 30 cycles of 18 s at 94 °C, 21 s at 50 °C and 3 min at 72 °C, and a final extension of 5 min at 72 °C.

For colony testing, primers specific for the M13F and M13R sites located on opposite sides of the multiple cloning site of the pCR-Blunt II-TOPO and pGEM-T Easy vectors were used. Plasmid-containing colonies were picked from LB/Agar plates directly into PCR master mix. Reactions for screening clones were performed under the following conditions: initial bacterial lysis/denaturation 2 min at 94 °C, 30 cycles of 18 s at 94 °C, 30 s at 50 °C and 60 s at 72 °C, and a final extension of 5 min at 72 °C. Reactions for full-length genome clones were performed under the following conditions: initial bacterial lysis/denaturation 2 min at 94 °C, 30 cycles of 18 s at 94 °C, 30 s at 50 °C and 3 min at 72 °C, and a final extension of 5 min at 72 °C.

#### Direct sequencing of PCR products and sequence analysis.

PCR amplicons were sequenced using either the M13F primer used in the colony screening or both Mt_F and Mt_R primers for species identification. Further sequencing of full-length genome clones was performed by primer walking. Sequencing was carried out using BigDye Terminator v3.1 (Applied Biosystems) according to the manufacturer’s instructions. Sequences were read by Edinburgh Genomics. Sequences, with vector nucleotides removed, were submitted for analysis using the NCBI (National Center for Biotechnology Information) Basic Local Alignment Search Tool for nucleotide or translated sequence homology (blastn or blastx).

Sequence analysis and assembly was performed using sse v1.1 (Simmonds, 2012). Protein coding sequences in complete genomes, identified using either sse v1.1 software or NCBI ORF Finder, were submitted for analysis using NCBI protein blast. Phylogenetic trees were reconstructed using maximum-likelihood methods as implemented in the mega6 software package (Tamura *et al.*, 2013).
